# Delayed Diagnosis of HDR (Hypoparathyroidism, Deafness, and Renal Dysplasia) Syndrome Presenting With Life-Threatening Hypercalcemic Crisis: A Case Report

**DOI:** 10.7759/cureus.105580

**Published:** 2026-03-21

**Authors:** İlker Çordan

**Affiliations:** 1 Division of Endocrinology and Metabolism, Department of Internal Medicine, Konya City Hospital, Hamidiye School of Medicine, University of Health Sciences, Konya, TUR

**Keywords:** acute kidney injury, barakat syndrome, gata3 mutation, hdr syndrome, hypercalcemic crisis, hypoparathyroidism, sensorineural hearing loss

## Abstract

Hypoparathyroidism, sensorineural deafness, and renal dysplasia (HDR) syndrome is a rare autosomal dominant disorder caused by pathogenic variants in the *GATA3* gene. Because the components of the clinical triad may emerge at different stages of life, isolated evaluation of findings by different specialties may lead to delayed syndromic diagnosis. In this report, we present the case of a 39-year-old female patient who was diagnosed with hypoparathyroidism following a hypocalcemia-related tetanic convulsion at the age of five years and was found to have sensorineural hearing loss during childhood. Although chronic kidney disease developed in adulthood, the multisystemic findings were not evaluated collectively, and a syndromic diagnosis was not established. The patient presented to the emergency department with altered consciousness, dehydration, and oliguria. Evaluation revealed severe hypercalcemia (20.7 mg/dL) and acute kidney injury on the background of chronic kidney disease (creatinine: 2.98 mg/dL; estimated glomerular filtration rate: 19 mL/minute/1.73 m²). Following urgent hemodialysis and intensive intravenous hydration, serum calcium levels normalized. However, complete recovery of renal function was not achieved. Genetic analysis identified a heterozygous pathogenic *GATA3* frameshift variant (c.431dup), which confirmed the diagnosis of HDR syndrome. This case emphasizes the importance of a syndromic approach and early genetic evaluation in patients presenting with hypoparathyroidism, hearing loss, and renal anomalies. In addition, careful individualization of calcium and active vitamin D replacement in individuals with reduced renal reserve appears critical to prevent life-threatening hypercalcemic complications.

## Introduction

Hypoparathyroidism, sensorineural deafness, and renal dysplasia (HDR) syndrome, also known as Barakat syndrome, is an extremely rare genetic disorder whose exact prevalence remains unknown; however, only a limited number of cases have been reported in the literature. The gene responsible for the syndrome is* GATA3*, located on the short arm of chromosome 10 [1,2]. *GATA3* encodes a dual zinc-finger transcription factor involved in embryologic development and plays a particularly crucial role in the development of the parathyroid glands, auditory system, and kidneys; therefore, pathogenic variants or haploinsufficiency of this gene lead to the characteristic triad of hypoparathyroidism, sensorineural hearing loss, and renal anomalies observed in HDR syndrome [3]. The wide spectrum of pathogenic *GATA3* variants and the highly variable clinical expression of HDR syndrome suggest that the disease is characterized by marked genotype-phenotype heterogeneity [2].

The classical clinical triad of HDR syndrome consists of hypoparathyroidism, sensorineural hearing loss, and renal anomalies; however, all three findings may not be present simultaneously in every patient at the time of diagnosis [4]. In the literature, only 53.7% of reported patients with *GATA3* mutations have been shown to present the complete HDR triad [5]. Diagnosis may be delayed because the clinical components may appear at different stages of life, and renal anomalies may remain asymptomatic for a prolonged period. This is one of the principal factors complicating early diagnosis of HDR syndrome.

There is no specific curative treatment for HDR syndrome, and clinical management is based on symptomatic and supportive therapy. Therefore, early diagnosis is of critical importance, particularly for close monitoring of renal function, individualization of calcium and active vitamin D replacement, early intervention for hearing loss, and provision of genetic counseling.

While hypocalcemia is the typical biochemical finding in HDR syndrome, severe hypercalcemia is not an expected manifestation of the disorder. In this case report, we aim to draw attention to the clinical heterogeneity of HDR syndrome and the serious complications that may arise from delayed diagnosis through the presentation of a patient with HDR syndrome who, despite childhood-onset hypoparathyroidism, remained undiagnosed until adulthood and presented with hypercalcemic crisis and acute kidney injury.

## Case presentation

A 39-year-old Turkish woman presented to the emergency department in February 2024 with complaints of progressive fatigue, altered consciousness, nausea and vomiting, excessive thirst, muscle weakness, and decreased urine output in recent days. At presentation, her general condition was moderate to poor. She was somnolent, and her cooperation was limited. Vital signs revealed a blood pressure of 95/60 mmHg, a heart rate of 115 beats/minute, a respiratory rate of 18 breaths/minute, a body temperature of 36.5 °C, and a peripheral oxygen saturation of 97% (on room air). Physical examination demonstrated decreased skin turgor and dry mucous membranes, findings consistent with marked dehydration. Neurological evaluation revealed altered consciousness, reduced proximal muscle strength, and hypoactive deep tendon reflexes. Her urine output was decreased.

Initial laboratory evaluation in the emergency department showed a serum total calcium level of 20.7 mg/dL. Serum phosphorus was 4.3 mg/dL, creatinine was 2.98 mg/dL, and estimated glomerular filtration rate (eGFR) was 19 mL/minute/1.73 m². Previous records indicated a baseline creatinine level of 1.3 mg/dL and an eGFR of 52 mL/minute/1.73 m². These findings were interpreted as acute kidney injury developing on the background of chronic kidney disease. Table 1 summarizes the patient’s baseline data from 2013, laboratory findings during the hypercalcemic crisis, and renal function and metabolic panel results from the first year after diagnosis.

**Table 1:** Changes in renal function and metabolic parameters over time Notes: “—”  indicates that no recorded data were available for the relevant period. eGFR was calculated using the CKD-EPI formula. Follow-up data correspond to evaluations performed during the first year after diagnosis. Protein/creatinine and albumin/creatinine ratios were obtained from spot urine samples. *Renal ultrasonography findings reflect the examination performed during the hypercalcemic crisis (February 2024). HDR, hypoparathyroidism, sensorineural deafness, and renal dysplasia; eGFR: estimated glomerular filtration rate; CKD-EPI: Chronic Kidney Disease Epidemiology Collaboration; TSH: thyroid-stimulating hormone

Parameter		First recorded data (August 2013)	Hypercalcemic crisis (emergency presentation in February 2024)	Follow-up (June 2025)
Glucose (mg/dL)		97	122	85
Creatinine (mg/dL)		0.71	2.98	1.6
eGFR (mL/dk/1.73 m²)		87	19	40
Uric acid (mg/dL)		3.3	6.8	7.3
Calcium (mg/dL)		6	20.7	8.8
Albumin (g/L)		44	37	45
Phosphorus (mg/dL)		6.9	4.3	3.9
Parathyroid hormone (pg/mL)		11.7	<1.2	<1.2
Protein/creatinine ratio (g/day)		—	317.61	510.2
Albumin/creatinine ratio (mg/L)		—	142.7	550.3
25-hydroxyvitamin D (ng/mL)		—	21	23.4
Sodium (mmol/L)		137	125	136
Potassium		4.3	4	4.7
Alkaline phosphatase (U/L)		94	214	50
Magnesium (mg/dL)		1.4	1.7	1.85
TSH (mIU/L)		2,55	1,75	3,75
Renal ultrasonography findings*	Renal ultrasonography revealed both kidneys in normal anatomical position, with sizes at the lower limit of normal. Bilateral grade 3 increased parenchymal echogenicity was observed, and corticomedullary differentiation was obliterated. No pelvicalyceal dilatation or calculi were detected, while parapelvic simple cysts were present in both kidneys.			

**Figure 1:**
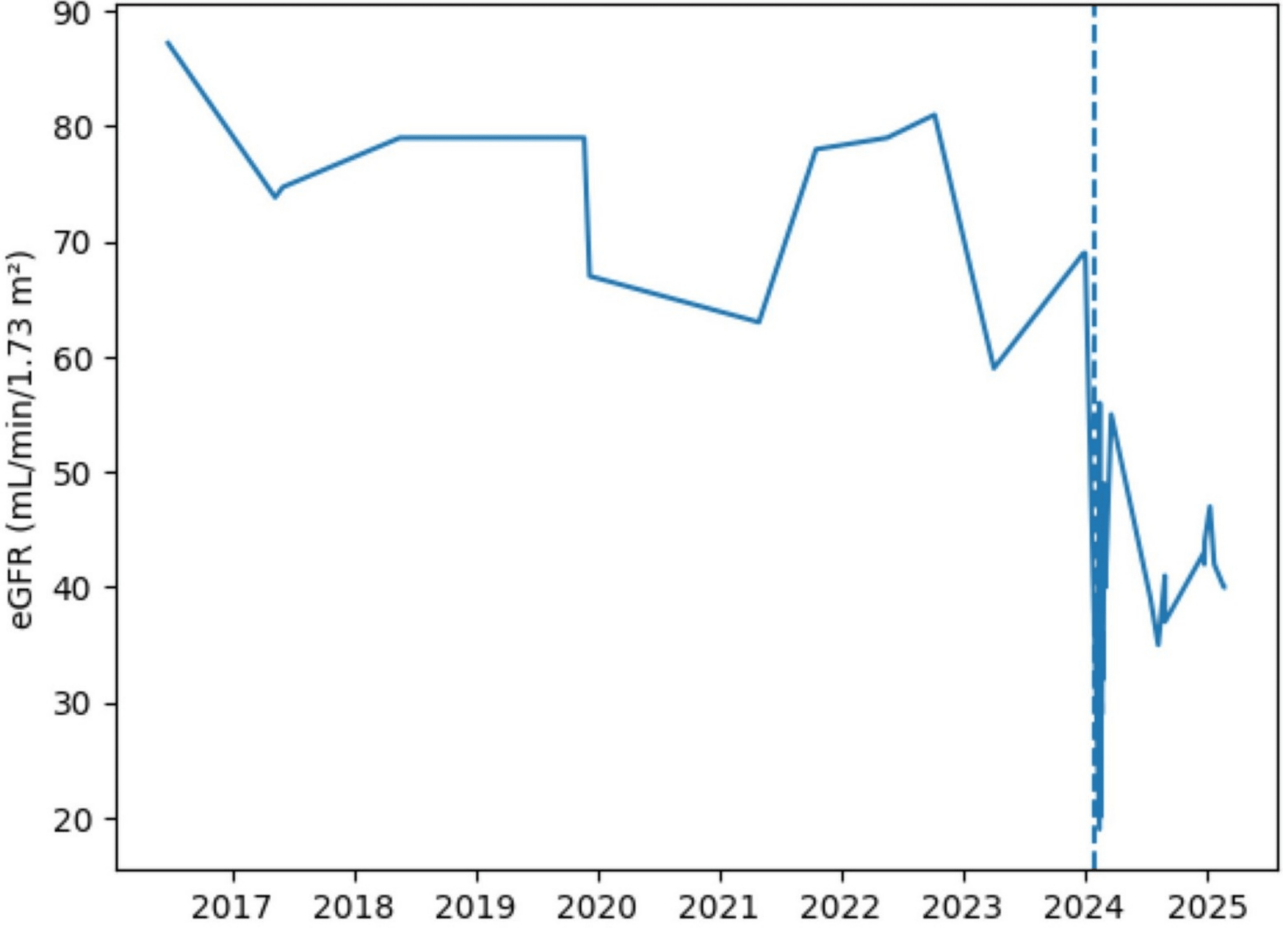
Longitudinal changes in estimated eGFR from 2016 to 2025. The dashed vertical line indicates the time of the severe hypercalcemic crisis in February 2024 (serum calcium: 20.7 mg/dL). A sharp decline in eGFR is observed during this period, followed by partial recovery after hemodialysis and intensive hydration therapy. However, renal function did not return to previous baseline levels, consistent with acute kidney injury superimposed on chronic kidney disease. eGFR: estimated glomerular filtration rate

When clinical and laboratory findings were evaluated together, the condition was interpreted as acute kidney injury characterized by altered consciousness, dehydration, and oliguria secondary to severe hypercalcemia, and the patient was admitted to the intensive care unit with an indication for urgent hemodialysis. After three sessions of hemodialysis and intensive intravenous hydration, there was a marked decrease in serum calcium levels and partial improvement in renal function. As illustrated in Figure 1, this acute event was associated with a sudden and pronounced decline in eGFR, followed by partial recovery after treatment. However, eGFR values did not return fully to baseline levels.

Further detailed history revealed that at the age of five years, the patient had presented with a tetanic convulsion due to hypocalcemia and was diagnosed with hypoparathyroidism during that period. After diagnosis, she received calcium and active vitamin D replacement therapy during childhood and adolescence; however, follow-up continuity was not maintained. It was learned that prior to the hypercalcemic crisis, the patient had been taking replacement therapy consisting of oral calcium carbonate providing approximately 2400 mg/day of elemental calcium together with vitamin D3 (1600 IU/day) and calcitriol 0.5 µg/day.

Sensorineural hearing loss was detected during childhood. Although a cochlear implant was placed, no clinically significant benefit was achieved, and the patient was using a hearing aid at the time of presentation to our hospital. It was also learned that she did not attend regular follow-up visits in adulthood. She subsequently developed chronic renal failure and was referred to a nephrology clinic; however, regular follow-up was again not maintained.

There was no history of consanguineous marriage in the family, and no similar phenotypic features were reported in the parents or first-degree relatives. However, the patient’s 11-year-old son had a history of intellectual disability, speech disorder, and epilepsy, and molecular analysis had not yet been performed to investigate a possible genetic etiology.

Considering the history of hypoparathyroidism at presentation, sensorineural hearing loss, and clinical and imaging findings related to chronic kidney disease (renal ultrasonography findings are presented in Table 1), a preliminary diagnosis of HDR syndrome was considered, and genetic analysis was planned. Genetic testing identified a heterozygous pathogenic frameshift variant in the *GATA3* gene (c.431dup; dbSNP ID: rs1588377948). This variant, which is associated with autosomal dominant HDR syndrome, results in a predicted loss-of-function effect and was classified as pathogenic according to the American College of Medical Genetics and Genomics (ACMG) criteria [6]. Based on these findings, the diagnosis of HDR syndrome was confirmed.

## Discussion

HDR syndrome is a rare genetic disorder that poses diagnostic challenges due to marked phenotypic heterogeneity and variable penetrance. The components of the clinical triad may not emerge simultaneously in every patient. Sensorineural hearing loss has been reported to have the highest penetrance and to most commonly present at an early age [5]. However, hypoparathyroidism is often the first clinical clue and may be evaluated for a prolonged period as an isolated endocrine disorder.

The diagnosis of HDR syndrome is primarily based on clinical findings. The coexistence of the three conditions constituting this syndrome, namely primary hypoparathyroidism, sensorineural hearing loss, and renal anomalies, is established through laboratory investigations, imaging modalities, and audiological assessments. Although genetic analysis is not mandatory for diagnosis, it is an important supportive tool for confirmation and clarification of the differential diagnosis [7]. In the current case, hypoparathyroidism began at the age of five years with a tetanic convulsion. The emergence of renal and otologic findings at different time points delayed comprehensive evaluation of the disease. The diagnosis was established only in adulthood during a hypercalcemic crisis when all components of the triad were considered together, suggesting that isolated assessment of multisystemic findings by different specialties may lead to delayed diagnosis in rare syndromes.

To date, approximately 200 cases of HDR syndrome have been reported in the literature, and a substantial proportion of these cases have been published in the last decade. This appears to be associated with increasing clinical awareness and advances in genetic diagnostic techniques [8]. To our knowledge, the current case represents the ninth reported case of HDR syndrome from Türkiye [9-14], including the patient previously reported by our research group [11]. Considering that some reports of rare diseases may have been published only as conference abstracts or in local journals, the actual number of cases may be higher. It is noteworthy that cases reported from Türkiye constitute approximately 10% of the total number of cases in the literature. Nevertheless, as illustrated by our patient, delayed syndromic diagnosis underscores the critical importance of a multidisciplinary and comprehensive approach for early diagnosis and long-term management in rare diseases.

Renal involvement, which may manifest across a broad clinical spectrum in HDR syndrome, is one of the principal determinants of long-term prognosis and may progress to end-stage renal disease [1,15]. The syndrome may be accompanied by renal dysplasia, unilateral or bilateral hypoplasia, cystic changes, vesicoureteral reflux, and electrolyte disturbances such as renal magnesium wasting [16]. However, it has been reported that renal involvement exhibits lower penetrance than hypoparathyroidism and sensorineural hearing loss, presents across a more variable age range, and may not become clinically apparent until advanced ages in some cases [17]. Supporting this, in our patient, we observed that the decline in eGFR beginning at 38 years of age had not been adequately recognized, and she presented with acute kidney injury developing on the background of chronic renal failure, accompanied by a hypercalcemic crisis.

Hypercalcemia and hypercalcemic crisis are not typical features of HDR syndrome. Although no clear acute trigger was identified in our patient, continuation of long-term calcium and active vitamin D replacement without dose adjustment in the setting of reduced eGFR may have contributed to a gradual increase in serum calcium levels. When decreased renal calcium excretion and increased intestinal absorption mediated by active vitamin D are considered together, an iatrogenic mechanism may be possible. It has also been previously reported that nephrocalcinosis may develop in patients with HDR syndrome, particularly in those receiving long-term calcium and active vitamin D replacement, and this may lead to deterioration in renal function [18]. Although cognitive involvement has been reported in HDR syndrome [1], raising the concern that the patient may have used her medications at higher than recommended doses, the absence of significant hypercalcemia in previous calcium monitoring makes this explanation less likely. Nevertheless, the available data are insufficient to determine the definitive etiology of the hypercalcemic crisis.

From a clinical perspective, this case highlights the importance of considering syndromic etiologies in patients presenting with early-onset hypoparathyroidism accompanied by renal or auditory abnormalities. In such patients, a systematic multidisciplinary evaluation involving endocrinology, nephrology, and otolaryngology is recommended. Although genetic analysis is not mandatory for diagnosis, the presence of hypoparathyroidism and sensorineural hearing loss, which are early manifestations of the syndrome, either separately or together, may raise suspicion, and genetic analysis for GATA3 mutations can help clarify and support the diagnosis. In addition, careful adjustment of calcium and active vitamin D therapy during long-term follow-up and regular monitoring of renal function are of great importance in preventing complications such as nephrocalcinosis or hypercalcemic crises and in planning more appropriate long-term management.

Although hypercalcemia resolved following intensive hydration and hemodialysis, failure of eGFR to return fully to baseline levels is consistent with acute kidney injury developing on the background of chronic kidney disease. This finding underscores the importance of individualizing replacement therapy and regularly monitoring renal function in patients with HDR syndrome. Careful reassessment of calcium and active vitamin D doses is critical to prevent potentially life-threatening hypercalcemic complications, particularly in individuals with reduced renal reserve.

## Conclusions

HDR syndrome is a rare autosomal dominant disorder characterized by marked phenotypic heterogeneity and variable age at onset. This case report describes a patient whose HDR syndrome was not recognized until adulthood despite a childhood diagnosis of hypoparathyroidism. It demonstrates that delayed diagnosis may lead to serious complications, including life-threatening hypercalcemic crisis and acute kidney injury developing on the background of chronic kidney disease. Therefore, early clinical awareness, genetic confirmation, regular multidisciplinary follow-up, and safe, individualized management of replacement therapy are of critical importance for the prevention of renal complications.
